# Evolutionary Adjustment of tRNA Identity Rules in Bacillariophyta for Recognition by an Aminoacyl-tRNA Synthetase Adds a Facet to the Origin of Diatoms

**DOI:** 10.1007/s00239-022-10053-5

**Published:** 2022-03-24

**Authors:** Gabor L. Igloi

**Affiliations:** Institute of Biology III, University of Freiburg, Freiburg, Germany

**Keywords:** Aminoacyl-tRNA synthetase, tRNA, Identity element, Bacillariophyta, Diatoms, Non-metazoan eukaryotic evolution

## Abstract

Error-free protein synthesis relies on the precise recognition by the aminoacyl-tRNA synthetases of their cognate tRNAs in order to attach the corresponding amino acid. A concept of universal tRNA identity elements requires the aminoacyl-tRNA synthetases provided by the genome of an organism to match the identity elements found in the cognate tRNAs in an evolution-independent manner. Identity elements tend to cluster in the tRNA anticodon and acceptor stem regions. However, in the arginine system, in addition to the anticodon, the importance of nucleotide A20 in the tRNA D-loop for cognate enzyme recognition has been a sustained feature for arginyl-tRNA synthetase in archaea, bacteria and in the nuclear-encoded cytosolic form in mammals and plants. However, nuclear-encoded mitochondrial arginyl-tRNA synthetase, which can be distinguished from its cytosolic form by the presence or absence of signature motifs, dispenses with the A20 requirement. An examination of several hundred non-metazoan organisms and their corresponding tRNA^Arg^ substrates has confirmed this general concept to a large extent and over numerous phyla. However, some Stramenopiles, and in particular, Diatoms (Bacillariophyta) present a notable exception. Unusually for non-fungal organisms, the nuclear genome encodes tRNA^Arg^ isoacceptors with C or U at position 20. In this case one of two nuclear-encoded cytosolic arginyl-tRNA synthetases has evolved to become insensitive to the nature of the D-loop identity element. The other, with a binding pocket that is compatible with tRNA^Arg^-A20 recognition, is targeted to organelles that encode solely such tRNAs.

**Supplementary Information:**

The online version contains supplementary material available at 10.1007/s00239-022-10053-5.

## Introduction

Aminoacylation is the enzymatic process by which amino acids are attached to their specific tRNAs during the first step of protein biosynthesis. It requires a virtually error-free recognition at two levels; the association of one of 20 aminoacyl-tRNA synthetases with its specific amino acid and the simultaneous identification of the cognate tRNA. Heterologous aminoacylation between macromolecules from different species foretold the existence of a common sequence within a given set of tRNAs isoacceptors that is responsible for their specific enzymatic association almost 60 years ago (Loftfield et al. 1968). However, such tRNA identity elements were experimentally verified only some 20 years later (Normanly et al. 1986; McClain and Foss 1988; Sampson et al. 1989; Schulman and Pelka 1989; Normanly and Abelson 1989). Extensive detailed investigation over many years of bacterial and yeast systems have provided good evidence that, despite some exceptions, for this rather limited selection of organisms a general set of universal rules governing the identity of each synthetase/tRNA pair exists (Giegé et al. 1998; Giegé and Eriani 2021). Further interspecies differences have provided some examples of a more idiosyncratic nature for tRNA recognition (Xue et al. 1993; Nameki et al. 1995; Stehlin et al. 1998). An evolutionary complication to the generalization had, however, arisen upon the emergence of eukaryotes since the synthetase/tRNA pair of the engulfed endosymbiont—the subsequent mitochondrion—possess distinct identity characteristics from the host (Kumazawa et al. 1989, 1991). Notably, the truncation of metazoan mitochondrial tRNAs and elimination of at least part of the anticipated identity elements provided an adequate minimal set of recognition sites (Ueda and Watanabe 1993; Fender et al. 2012) and opened a whole range of exceptions to the convenient universal identity element concept (Lovato et al. 2001; Yuan et al. 2011; Fender et al. 2012; Igloi and Leisinger 2014; Zeng et al. 2019) leading in some instances even to natural codon reassignments (Ling et al. 2014). In metazoans, the appearance of mitochondria and their encoded tRNAs, therefore, required the retention of both nuclear-encoded cytosolic aminoacyl-tRNA synthetase and nuclear-encoded mitochondrial aminoacyl-tRNA synthetase with distinct recognition potentials.

Identity elements tend to cluster in the tRNA anticodon and acceptor stem regions (Carter and Wolfenden 2016). However, in the arginine system, the importance of nucleotide A20 in the tRNA D-loop for cognate enzyme recognition was first established both in vivo (McClain and Foss 1988) and in vitro for *E.coli* (Tamura et al. 1992) and has been a sustained feature also for arginyl-tRNA synthetase for archaea, (Mallick et al. 2005) and the cytosolic form in mammals (Guigou and Mirande 2005) and plants (Aldinger et al. 2012). Its role in binding to the enzyme has been examined in crystallographic detail for Eubacteria (*Escherichia coli*: Stephen et al. 2018), Archaea (*Pyrococcus horikoshii*; Konno et al. 2009) and a docking model for *Thermus thermophilus* has been presented (Shimada et al. 2001). The variable pocket encompassing the tertiary structure of the D and T loops of tRNA^Arg^ constitutes an essential feature in its recognition by arginyl-tRNA synthetase. In these three microorganisms, aminoacylation is strictly dependent on the presence of adenosine at position 20 (A20) in tRNA^Arg^. In contrast, *Saccharomyces cerevisiae*, often taken as a good model for eukaryotic systems, possesses a cytosolic arginyl-tRNA synthetase that is conspicuous in not requiring this A20 for recognition (Sissler et al. 1996) and, indeed, is indifferent to the nature of the base at that position.

Phylogenetic analysis has revealed that the arginyl-tRNA synthetase of *S. cerevisiae* is the product of an ancestral mitochondrial gene that, after migration to the nucleus and following duplication, has replaced the gene for the host cytosolic form (Karlberg et al. 2000; Brindefalk et al. 2007). Therefore, the identity elements documented for the *S.cerevisiae* arginyl-tRNA synthetase, and in particular, the indifference to the base at position 20 in tRNA, correspond to those found in mitochondrial tRNAs having canonical cloverleaf structures, as in Fungi. An alignment of derived amino acid sequences of arginyl-tRNA synthetases over a wide phylogenetic range has uncovered sequence domains specific to the nuclear-encoded cytosolic and to the nuclear-encoded mitochondrial arginyl-tRNA synthetases, respectively (Igloi 2020a) which one can use to classify the ancestral source of the corresponding gene (Table 1). Using this classification and a knowledge of the corresponding identity elements (Aldinger et al. 2012) it is possible to follow the co-evolutionary development of the macromolecules.

**Table 1:** Signature sequences used to classify arginyl-tRNA synthetase to their ancestral cytosolic or mitochondrial origin and their associated tRNA identity elements

Arginyl-tRNA synthetase type	Classification based on:		
	N-term D-loop binding domain	KMSK catalytic domain	tRNA Identity Element at D-loop Position 20
Cytosolic	“GDYQ”	“KFKTR”	A
Mitochondrial	Poorly discernible “GDYQ”	“5∆MSTR” or “MSSR”	U, C, or A

A concept of universal identity elements would require that the enzyme(s) provided by the genome of an organism match the identity elements found in the cognate tRNAs in an evolution-independent manner. In its simplest form it would be expected that cytosolic-type arginyl-tRNA synthetase has a requirement for tRNA^Arg^-A20, irrespective of the cellular compartment encoding the tRNA, as, for example, in plants. In contrast, the mitochondrial-type enzyme could recognize a tRNA^Arg^ with any nucleotide at position 20, again in a subcellular-independent manner, as, for example, in Fungi. The validity of such universal identity elements has previously been confirmed (Igloi 2021) for the relatively restricted sample size of amitochondrial organisms (Makiuchi and Nozaki 2014). Whether it also applies to a much more complex system of multi-organelle species, possibly with multiple arginyl-tRNA synthetase genes, has been examined here.

Recognition rules for non-metazoans are poorly understood but have been examined in an Apicomplexan tyrosine system from Plasmodium (Cela et al. 2018). In that organism a minimalistic set of elements compared with the evolutionarily conserved positions was detected. Taking this observation as an indication that non-metazoans might be a source of unconventional identity rules, a screening of arginyl-tRNA synthetases within the phylogenetic groups of non-metazoan/non-fungal eukaryotes was undertaken.

## Results

A set of 404 derived protein sequences corresponding to one or more distinct arginyl-tRNA synthetase genes from 264 non-metazoan, non-fungal eukaryote species and covering 32 taxonomic groups in the National Center for Biotechnology Information (NCBI) taxonomic classification were assembled (Igloi 2022). Species possessing a mitosomal organelle have been discussed previously (Igloi 2021) and were not included. Sequences from individual taxonomic groups were visually inspected in order to classify them into the cytosolic or mitochondrial category (Online Resource 1) using the sequence features described previously (Igloi 2020a) (Table 1). For non-metazoans, the elements distinguishing the forms were not as clear-cut as in the case of Eumetazoans. In particular the five amino acid-deletion in the characteristic mitochondrial 5∆MSTR motif (Igloi 2020a) was not always present. Instead, the cytosolic KFKTR-like motif (corresponding to the conserved Class I aminoacyl-tRNA synthetase KMSK region (Sekine et al. 2001)) was frequently replaced by MSSR, or similar. More reliably, the cytosolic N-terminal GDYQ-motif becomes barely detectable in the mitochondrial form. However, in some cytosolic instances, the GDYQ-sequence was also considerably degraded (Online Resource 2). For questionable placement, an alignment with a clustering of distinct forms of the sequences could provide clarification.

No tRNA sequence data (either cytosolic or organelle) for 61 of these species could be extracted from the databases and could not be used for identity analysis but the protein sequences were nevertheless included in the alignments to facilitate classification. In order to detect robust and systematic evidence for a molecular mechanism of tRNA recognition by alternative or unconventional co-evolution, 931 tRNA^Arg^ isoacceptors; (568 nuclear; 169 mitochondrial; 194 plastid) from species corresponding, where possible, to the source of the enzymes, were compiled (Igloi 2022). Examination of the enzymes from 203 species for which tRNA data were available showed that there were 40 potential candidates for identity element erosion. As some candidates were scattered within phyla containing otherwise canonical synthetase/tRNA pairs and the identity deviation relies on the nature of a single tRNA base, their corresponding cytosolic and organelle tRNAs were examined in more detail. Some questionable identity erosion could be eliminated taking into account mis-annotation, suspect sequence data or potential contamination of environmental samples. Some examples of potential alternative recognition processes were detected in data from phyla represented by single or only few species and are, therefore, not suitable for coming to evolutionary generalizations. However, they have been listed and analysed (Online Resource 2).

The polyphyletic group of photosynthetic organisms (including Dinophyceae, Cryptophyta, Euglenophyta, Haptophyta, Rhodophyta, Eustigmatophyceae, Pelagophyceae, Phaeophyceae, Xanthophyceae and Chlorophyta) contribute 103 algal species and 136 arginyl-tRNA synthetase sequences. All enzymes are of the cytosolic-type and are associated with tRNAs having A20 in all sub-cellular compartments. They may be destined for recognizing all cellular tRNAs (as in higher plants (Duchêne et al. 2005)) or in the case of pairs of different genes in some taxa (e.g. Phaeophyceae), have distinct subcellular targets.

Of these photosynthetic phyla, Pelagophyceae, Phaeophyceae and Xanthophyceae belong to the clade of Stramenopiles. However, within the Stramenopiles, the phylum Bacillariophyta provides a remarkable and consistent exception to the presumed recognition of universal tRNA identity elements. Bacillariophyta (Diatoms) are represented in the databases by 51 arginyl-tRNA synthetase sequences from 26 species (Table 2) and correspondingly 97 tRNA^Arg^ isoacceptors (Igloi 2022), encoded by the nucleus, mitochondrion or plastid, from 18 species. They provide a convincing example of a novel evolutionary divergence from the previously held concept of how arginyl-tRNA synthetase deals with distinct identity elements in its cognate tRNAs.

**Table 2:** Bacillariophyta species for which arginyl-tRNA synthetase gene products were compiled

Species	Nature of base at position 20 in tRNA		
	Cyto	Mito	Plastid
***Asterionella formosa 1, Asterionella formosa 2***	A, C, U	A	A
***Asterionellopsis glacialis1, Asterionellopsis glacialis2***	C, U		A
***Chaetoceros neogracilis1, Chaetoceros neogracilis2***	A, U		A
***Conticribra weissflogii1, Conticribra weissflogii2***	C		A
*Corethron pennatum1, Corethron pennatum2*			
***Coscinodiscus wailesii1, Coscinodiscus wailesii2***	U		A
***Cylindrotheca closterium1, Cylindrotheca closterium2***	U	A	A
*Dactyliosolen fragilissimus1, Dactyliosolen fragilissimus2*			
***Ditylum brightwellii1, Ditylum brightwellii2***	C, U		
***Fistulifera solaris1, Fistulifera solaris2***	C, U	A	A
***Fragilariopsis cylindrus 1, Fragilariopsis cylindrus 2***	C, U	A	A
***Guinardia flaccida1, Guinardia flaccida2***			A
***Leptocylindrus danicus1, Leptocylindrus danicus2***			A
*Minidiscus sp*			
*Minutocellus polymorphus1, Minutocellus polymorphus2*			
***Navicula sp1, Navicula sp2***	C	A	
***Nitzschia sp1, Nitzschia sp2***	U	A	A
***Odontella aurita***		A	A
***Phaeodactylum tricornutum 1, Phaeodactylum tricornutum 2***	U	A	A
***Pseudo-nitzschia multistriata, Pseudo-nitzschia fraudulenta***	U	A	A
***Skeletonema marinoi1, Skeletonema marinoi2***	U	A	
*Stephanopyxis turris1, Stephanopyxis turris2*			
*Synedra sp1, Synedra sp2*			
*Synedropsis cf. recta*			
***Thalassiosira pseudonana 1, Thalassiosira pseudonana 2***	C, U	A	A
*Tryblionella compressa1, Tryblionella compressa2*			

Those species, which on the basis of available tRNA data, require a relaxed recognition property, are highlighted in bold. For these, where available, the nature of the base at position 20 in tRNA^Arg^ is given. Unbolded correspond to species with unavailable tRNA records

*Cyto* cytosolic, *Mito* mitochondrial

**Fig. 1:**
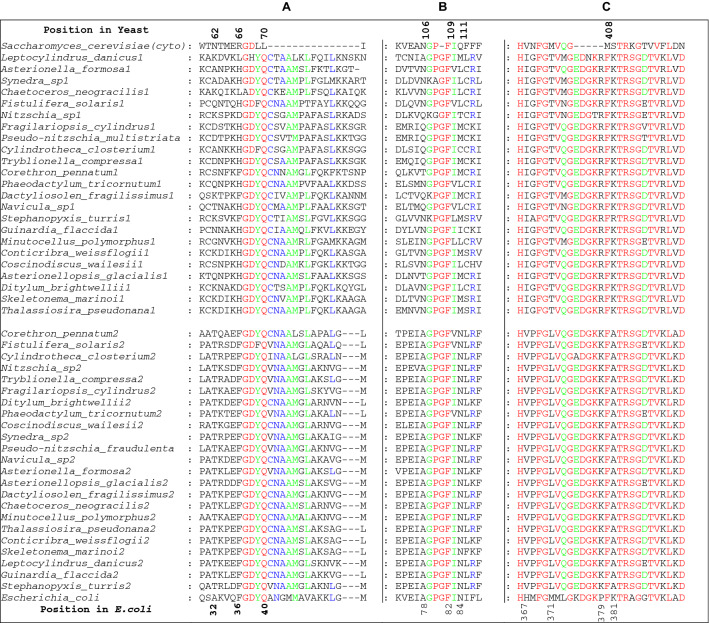
Alignment of Bacillariophyta arginyl-tRNA synthetase tRNA D-loop binding regions (Panels A and B) and of the conserved “KMSK” catalytic region (Panel C). For orientation, segments from the *E.coli* and *S.cerevisiae* enzymes and the numbering of their critical residues is given. The species order is that given by the CLUSTAL Ω alignment and results in the two distinct clusters. Amino acid identity is shown as Red > 95%; Green 80–95%, Blue 60–80% (Color figure online)

**Fig. 2:**
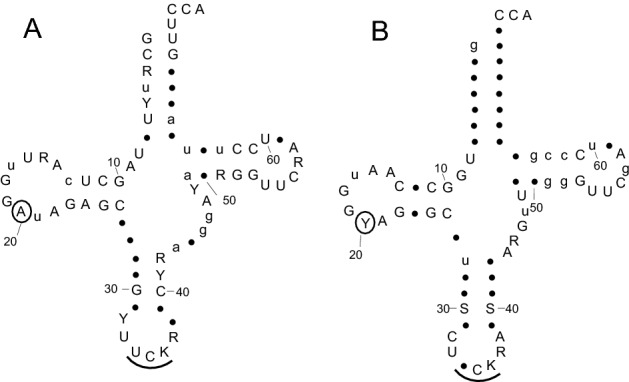
Cloverleaf depiction of tRNA^Arg^ isoacceptors that either require recognition by an arginyl-tRNA synthetase, which is indifferent to the nucleotide at position 20, or possessing A20 as a potential identity element. Shown are consensus sequences derived for all available Bacillariophyta tRNA^Arg^ isoacceptors encoded either by the mitochondrion, 22 sequences (**A**) or by the nucleus, 33 sequences (**B**). Nucleotide 20 is circled in each case and the anticodon is underlined. Nucleotides are given according to the IUPAC convention; Y, pyrimidine; R, purine; S, G or C; K, G or U. For recording consensus sequences, upper case denotes > 95% identity, lower case denotes 80–95% identity. • represents greater variability. Nucleotide numbering follows the convention established for tRNAs (Sprinzl et al. 1998)

In all but three species, two genes for the enzyme have been revealed by BLAST searches [the missing genes are likely to be due to gaps in the respective genome assemblies or transcriptome (TSA) databases)] (Table 2). Both gene products are clearly of the cytosolic-type, possessing conserved GDYQ and KFKTR motifs (Fig. 1, Panels A and C). The plastids are said to originate from Rhodophyta (Falkowski et al. 2004) and accordingly all encoded tRNA^Arg^ isoacceptors (43 isoacceptors from 15 species) have retained the algal nucleotide A20. The encoded mitochondrial tRNA^Arg^, without exception (22 isoacceptors from 11 species) also carry the A20 (Fig. 2A) identity element for recognition by the cytosolic form of the enzyme. However, the available nuclear-encoded tRNAs from 15 species possess C20 and/or U20 (Fig. 2B) without exception (Table 2). This results in the paradox of one form of the cytosolic enzyme needing, atypically, to be indifferent to the nature of the base at position 20 and requires a more detailed examination.

**Fig. 3:**
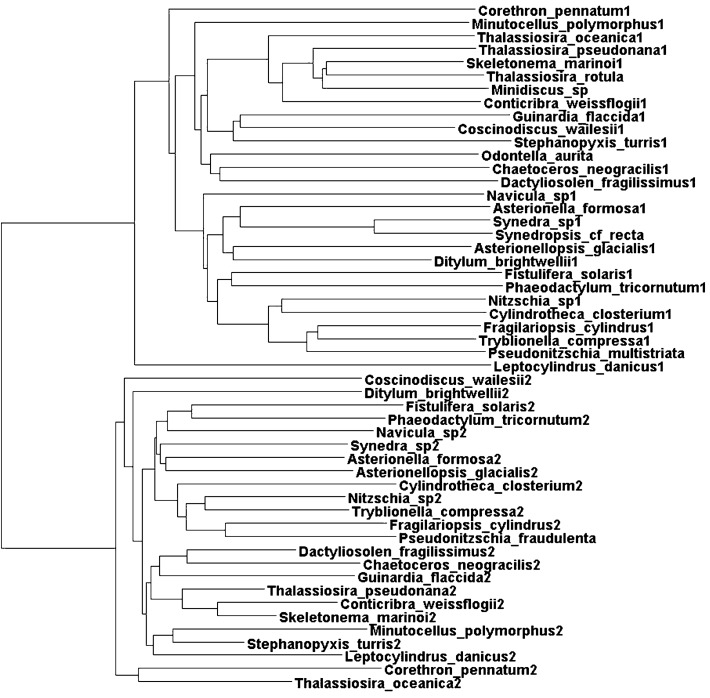
Clustering of Bacillariophyta arginyl-tRNA synthetase gene products in two groups. Alignments were performed by CLUSTALΩ and the resulting phylogenetic tree depicted in DENDROSCOPE (Huson and Scornavacca 2012)

**Fig. 4:**
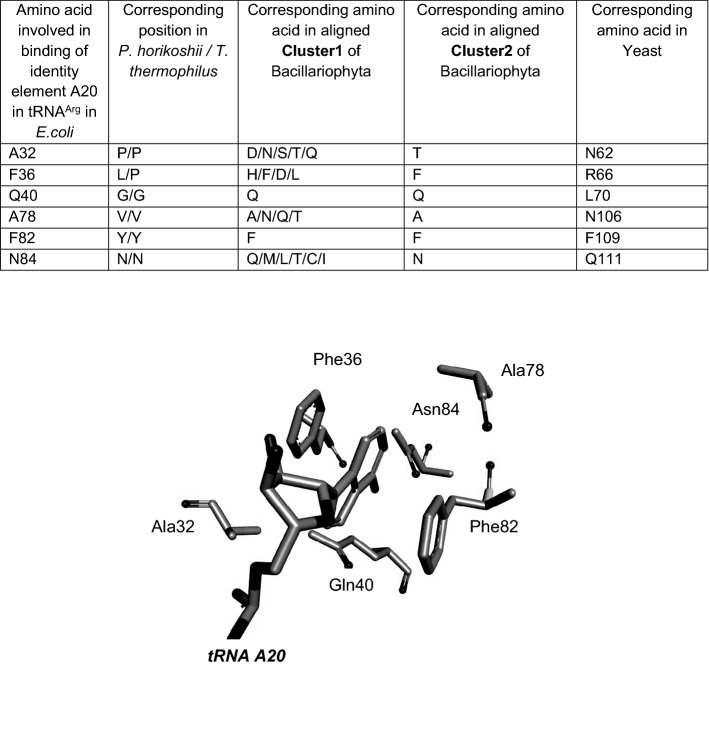
A comparison of the Bacillariophyta clusters of arginyl-tRNA synthetases with bacteria and yeast. Amino acids involved in the binding of the tRNA variable pocket that includes the identity element at position 20 are listed and the structure of the binding pocket in the *E.coli* crystallographic arginyl-tRNA synthetase/tRNA complex (Stephen et al. 2018), extracted from the Protein Data Bank (5YYN) and depicted in DISCOVERY STUDIO 4.0 is shown

Sequence alignment of the proteins using species which provided data from two genes, resulted in two well-defined phylogenetic clusters each containing one of the gene products which were then designated arbitrarily as Sequence1 or Sequence2 depending on their grouping in the clusters (Fig. 3). In order to determine which cluster was responsible for recognizing A20-containing tRNA^Arg^ isoacceptors, attention was focussed on the amino acids that have been defined crystallographically as being involved in the binding of the tRNA variable pocket (including position 20) in yeast (Delagoutte et al. 2000), bacteria (Shimada et al. 2001; Stephen et al. 2018) and archaea (Konno et al. 2009). Numerous amino acids make up the D-loop binding pocket and these can vary between organisms (Fig. 4). Furthermore, the N-terminal domain which comprises this pocket adopts a different orientation in bacteria compared to yeast (Bi et al. 2014). In *E.coli* A20 in the D-loop is locked into the enzyme by a mesh of H-bonds involving F^36^, Q^40^, A^78^ and N^84^ and stacking with F^82^ (Stephen et al. 2018) (Fig. 4). A^32^ in *E.coli* corresponds to P^29^ in *T.thermophilus* which forms a van der Waals interaction with A20 (Shimada et al. 2001) and is conserved as T in all Protein2 members (Fig. 1 Panels A and B). Compared to *E.coli* A^78^, the analogous N^106^ of yeast contacts tRNA D20, but mutation N^106^A in yeast is viable and the kinetic constants of this mutation, as well as F^109^A and Q^111^A in yeast arginyl-tRNA synthetase are the same as those on the wild-type (Geslain et al. 2003). This shows that the interaction between the N-terminal domain of *S. cerevisiae* arginyl-tRNA synthetase and the D-loop of tRNA^Arg^ are not important for the specific interaction. Indeed, the arginine-accepting activity was not decreased by the replacement of tRNA C20 by A in an *S. cerevisiae* tRNA^Arg^_UCU_ species (Guigou and Mirande 2005). Thus, structural changes in the protein from the strictly A20-binding *E.coli*-like domain to the yeast-like relaxed binding network can result in indifference to position 20 of the tRNA.

In Bacillariophyta, the Sequence2 representatives were, in general, more *E.coli*-like than Sequence1 proteins (Fig. 4). Within the conserved FGDYQ motif in cytosolic enzymes, the F corresponding to F^36^ in *E.coli* motif, which is in contact with A20, is conserved throughout the Sequence2 cluster and is replaced frequently, but not uniformly by H in Sequence1. On the other hand, *E.coli* Q^40^ of GDYQ, close to A20 (Stephen et al 2018) is present in all Gene1 as well as in Gene2 products (Fig. 1, Panel A). As noted, position 106 in yeast (*E.coli* 78) is replaced by a small residue (here, A) to accommodate the adenosine ring (Geslain et al. 2003) in Gene2 products, whereas N/Q is retained in Gene1 products. Of the other residues in this domain, *E.coli* F^82^ which is stacked on A20 and interacts with D20 in yeast is conserved throughout Bacillariophyta. In *E.coli*, N^84^ would appear to play a central role in A20 recognition. The corresponding amino acid N^79^ in *T.thermophilus* has been replaced by a selection of different amino acids (Sekine et al. 2001) and revealed that N^79^D could aminoacylate both tRNA^Arg^A20 and tRNA^Arg^G20 (although G20 occurs very rarely, if at all, in natural tRNA^Arg^ isoacceptors). Interestingly, the N^79^Q mutation, which would correspond to the Q^111^ position in yeast proved to be inactive with all position 20 variants, indicating that the importance of N^79^ relies on its associated architecture. This crucial D-loop recognition which is guided by N^84^ (Stephen et al. 2018) is conserved throughout Gene2 products of Bacillariophyta but is highly variable in the other cluster (Fig. 1, Panel B).

Although the domain KMSK that is typical of Class I aminoacyl-tRNA synthetases is not involved tRNA binding, it is vital in catalysis (Sekine et al. 2001) and its alignment (Fig. 1, Panel C) demonstrates its conserved nature. It is worth noting, however, that even within this highly conserved sequence segment, conserved differences between Sequence1 and 2 exist. Examples are V/I at position *E.coli* 367, T/L at 371, R/K at 379 and K/A at 381. Such conserved divergence is found at several sites throughout the protein as in cluster-specific deletions (Panel A). The compilation also shows the nature of the 5∆MSTR motif found in yeast (position 408) and which is typical of nuclear-encoded mitochondrial arginyl-tRNA synthetases (Igloi 2020a). It is evident that none of the Bacillariophyta enzymes correspond to this motif but, as indicated additionally by the N-terminal GDYQ feature both Sequences1 and 2 all fulfil the requirements to be of the cytosolic form. Nuclear-encoded tRNA^Arg^ in Bacillariophyta are, without exception within these samples, characterized by having U20 or C20 isoacceptors (Table 2). Conventional identity rules would require these to be recognized by yeast-like, mitochondrial enzymes. However in the absence of this enzyme type, one of these two cytosolic enzymes must, atypically, be insensitive to the nature of the nucleotide at position 20.

From the examination of the D-loop binding domain of bacterial arginyl-tRNA synthetases (above) Protein2 clearly fulfils the requirements for A20 binding, whereas Protein1 lacks some of the essential interacting amino acids (F^36^, A^78^, N^84^ in *E.coli*). In order to assess the likelihood of proteins of group 2 being involved in protein synthesis involving organelle-encoded tRNA-A20 recognition, their targeting potential was examined with five different prediction algorithms, including DEEPLOC 1.0, which has proved to be robust for mitochondrial proteins in diatoms (Cainzos et al. 2021), (Table 3).

**Table 3:** Organelle targeting prediction for Bacillariophyta arginyl-tRNA synthetase Gene1 and Gene2 products

Organism	Algorithm					Number of algorithms giving organelle location
	Busca	Tagetp	Deeploc	Mulocdeep	Hectar	
*Asterionella formosa1*	c	c	c	c	c	
*Asterionella formosa2*^*a*^	c	c	O	O	c	2
*Asterionellopsis glacialis1*	c	c	c	c	c	
*Asterionellopsis glacialis2*	O	O	O	O	O	5
*Chaetoceros neogracilis1*	c	c	c	c	c	
*Chaetoceros neogracilis2*	O	O	O	O	O	5
*Conticribra weissflogii1*	c	c	c	c	c	
*Conticribra weissflogii2*	O	O	O	O	O	5
*Corethron pennatum1*^*b*^	O	O	O	O	c	
*Corethron pennatum2*	O	c	c	O	c	2
*Coscinodiscus wailesii1*	c	O	c	c	c	
*Coscinodiscus wailesii2*	O	O	O	O	O	5
*Cylindrotheca closterium1*	c	c	c	c	c	
*Cylindrotheca closterium2*	O	O	O	O	O	5
*Dactyliosolen fragilissimus1*^*b*^	c	c	c	c	c	
*Dactyliosolen fragilissimus2*^*b*^	c	c	c	c	c	0
*Ditylum brightwellii1*	c	c	O	c	c	
*Ditylum brightwellii2*	c	O	O	O	O	4
*Fistulifera solaris1*	c	c	c	c	c	
*Fistulifera solaris2*	O	O	O	O	O	5
*Fragilariopsis cylindrus1*	c	c	c	c	c	
*Fragilariopsis cylindrus2*	O	O	O	O	c	4
*Guinardia flaccida1*	c	c	c	c	c	
*Guinardia flaccida2*	O	O	O	O	O	5
*Leptocylindrus danicus1*	c	c	c	c	c	
*Leptocylindrus danicus2*	O	c	O	O	O	4
*Minutocellus polymorphus1*	c	c	c	c	c	
*Minutocellus polymorphus2*	O	O	O	O	O	5
*Navicula sp1*	c	c	c	c	c	
*Navicula sp2*	O	O	O	O	O	5
*Nitzschia sp1*	c	c	c	c	c	
*Nitzschia sp2*	O	O	O	O	O	5
*Phaeodactylum tricornutum1*	c	c	c	c	c	
*Phaeodactylum tricornutum2*	O	O	O	O	O	5
*Pseudo-nitzschia multistriata(1)*	c	c	O	c	c	
*Pseudo-nitzschia fraudulenta(2)*	O	c	c	O	O	3
*Skeletonema marinoi1*^*b*^	c	c	O	O	c	
*Skeletonema marinoi2*^*b*^	c	c	c	c	c	0
*Stephanopyxis turris1*^*b*^	c	c	c	c	c	
*Stephanopyxis turris2*^*b*^	c	c	c	c	c	0
*Synedra sp1*	c	c	c	c	c	
*Synedra sp2*	O	O	O	O	O	5
*Thalassiosira oceanica1*	c	c	c	c	c	
*Thalassiosira oceanica2*^*a*^	O	c	c	c	c	1
*Thalassiosira pseudonana1*	c	c	c	c	c	
*Thalassiosira pseudonana2*^*a*^	c	c	c	c	c	0
*Tryblionella compressa1*	c	c	c	c	c	
*Tryblionella compressa2*	O	O	O	O	O	5

Predictions were performed with the algorithms indicated *c* cytosol, *O* organelle

Numbers in brackets refer to different species from the same genus

^a^Derived from genomic sequence; N terminus not confirmed

^b^TSA encodes a protein which is N-terminally incomplete

Despite the relative uncertainty of predictions and the evident N-terminal extension in the Protein1 group (Igloi 2022), the general tendency is for the Protein2 group to become transported in the organelles (Table 3). Some exceptions are apparent but these concern sequences whose N termini are either not complete or are ill-defined from genomic assemblies. One can, therefore, with a degree of reliability maintain that the enzymes whose D-loop binding characteristics are in accordance with tRNA-A20 recognition, are targeted to the cellular location harbouring such tRNAs. In contrast group 1 enzymes are retained in the cytosol and have evolved to abstain from using position 20 as a recognition element.

## Discussion

Rules governing the specific recognition of tRNAs by their aminoacyl-tRNA synthetases have accumulated over the past decades for all aminoacyl-tRNA synthetase/tRNA pairs. Although some exceptions have been recognized and by no means all taxonomic groups have been investigated, in view of their conservation, it is generally accepted the some key identity elements embedded in the structure of tRNA have been maintained throughout evolution and encompass the Tree of Life (Giegé and Eriani 2021). Nevertheless, exceptions, in particular involving metazoan mitochondrial systems and in the case of some much less-studied taxonomic groups such Apicomplexans have been noted (Giegé and Eriani 2021).

A frequently cited apparent exception that has been known for decades (Benzer and Weisblum 1961; Giegé et al. 1998) is the distinct recognition mechanism by arginyl-tRNA synthetase from *E. coli* and from *S. cerevisiae*. Both enzymes rely on the presence of C35-U/G36 in the anticodon but the former is, in addition, strictly dependent on the major identity element adenosine at position 20 in the tRNA D-loop. The yeast enzyme is characteristically indifferent to the base in the D-loop. However, a frequently overlooked aspect (McShane et al. 2016) is that the yeast cytosolic enzyme is, in fact, derived from an ancestral mitochondrial gene that migrated to the nucleus and, after duplication, replaced the gene for the host cytosolic form (Karlberg et al. 2000; Brindefalk et al. 2007). Therefore, the tRNA binding by the modern yeast cytosolic enzyme [or the enzyme from Fungi, in general (Igloi 2020a)] despite being propagated in the literature, is not comparable to the bacterial or indeed to cytosolic eukaryotic enzymes that were derived from bacteria after the endosymbiotic evolution of mitochondria.

An alignment of several hundred arginyl-tRNA synthetase sequences from numerous taxonomic sources (Igloi 2020b) revealed that one can distinguish between the cytosolic and the mitochondrial enzyme type by characteristic sequence motifs (Igloi 2020a). Using these markers the arginyl-tRNA synthetase of any organism can be matched to the identity elements presented by its cognate tRNA. In the simplest application, an examination of amitochondrial species revealed the single arginyl-tRNA synthetase encoded by the genome always corresponded to the identity elements found in the cytosolic tRNA^Arg^ irrespective of whether the enzyme was of the cytosolic or mitochondrial class. It was proposed that in amitochondrial organisms the choice between loss and retention of one type of arginyl-tRNA synthetase depended on the nature of the nucleotide at position 20 of the cognate tRNA (Igloi 2021).

An extension of this analysis to include organisms possessing subcellular genetic compartments in order to determine whether this principle is valid in more complex systems, has now been performed. Attention has been focussed on less well-studied non-metazoans/non-fungi whose subcellular tRNAs are of a canonical structure and would be expected to follow conventional identity rules.

Although the supposition that nucleotides that are conserved at given sites can be equated with identity elements needs to be validated experimentally in each case, the overall trend appeared to substantiate the concept of cytosolic-type arginyl-tRNA synthetases requiring tRNA-A20, whereas mitochondrial-type enzymes were needed to recognize tRNA-N20. Of the 264 species providing sequence data, and excluding 61 species without associated tRNA^Arg^ data, 168 from 26 phyla were found to match the macromolecules according to the anticipated recognition rules (Online Resource 1). Forty appeared to diverge from the identity framework in having cognate tRNAs without the A20 identity element required by the cytosolic arginyl-tRNA synthetase. Despite an intensive manual BLAST-supported search of genomic, transcriptome and specialized databases, numerous non-metazoan taxa were only represented by individual or few species making definitive statements regarding taxonomy-dependent identity of these unrealistic. Some proved to be questionable due to the problems of potential host contamination of environmental samples, as has been documented for other studies (Borner and Burmester 2017). Others were the result of mis-annotation (e.g. mitochondrial tRNAs being classified as nuclear encoded during total transcriptome analysis). However, individual deviations that could not be ignored as artefacts may require experimental verification. These are discussed in detail in Online Resource 2.

Nevertheless, a striking exception to the presumed universal identity rules was found to be maintained throughout Diatoms (Bacillariophyta). Of the 26 species for which arginyl-tRNA synthetase sequences could be recovered by BLAST searches, 21 proved to have two distinct gene products which were all ascertained to originate from ancestral nuclear genes, on the basis of characteristic signature sequences (Igloi 2020a). The two gene products gave rise to two well-defined alignment clusters. This, in itself, would not be a cause for concern since dual targeting has been reported in Diatoms (Gile et al. 2015) as well as in plants (Duchêne et al. 2005) where the second gene product is destined for import into both organelles. However, an examination of the substrate tRNAs encoded in the nuclear genome and in the organelle genomes, showed that the cytosolic tRNAs were not compatible with the hitherto accepted universal identity elements. The major element A20 in the D-loop, which has so far been a consistent feature of cytosolic arginyl-tRNA synthetase recognition, had been replaced by C20 or U20. To allow recognition of such tRNAs the cytosolic-type of arginyl-tRNA synthetase would have required the evolution of a mitochondrial-type of tRNA binding structure, as seen e.g. in *S. cerevisiae*, whose arginylation activity is insensitive to the nature of the base at position 20 (Sissler et al. 1996). A comparison of the Bacillariophyta enzymes with the crystal structures (Delagoutte et al. 2000; Konno et al. 2009; Stephen et al. 2018), which pinpointed the amino acids responsible for forming the D-loop binding pocket, showed that for each Diatom one gene product carried conserved amino acid changes which would be compatible tRNA recognition in the absence of A20. One should, however, also be aware that in Diatom cytosolic tRNAs additional, currently unrecognized, identity elements may have emerged as auxiliary elements, as was the case in metazoan mitochondrial arginyl-tRNA synthetase (Igloi and Leisinger 2014). Consistent with the notion that such an altered enzyme was destined for cytosolic protein synthesis, was the finding that the second alignment cluster was predicted to be targeted to the organelles. The proteins in this group have all the structural properties of a tRNA-A20-binding arginyl-tRNA synthetase which is required for the mitochondrial- and plastid-encoded tRNA^Arg^ isoacceptors.

Although the nature of the ancestral eukaryotic host prior to endosymbiotic acquisition of the “red” plastid (Falkowski et al. 2004; Sims et al. 2006; Benoiston et al. 2017) is not well-defined, its nucleus either possessed or gained tRNA genes through horizontal transfer, which are transcribed to U or C at position 20. The retention of U/C20 bases in the nuclear-encoded tRNAs has required either the perpetuation of the corresponding ancestral aminoacyl-tRNA synthetase from the heterotrophic host or a complementary re-adjustment within the structure of a duplicated cytosolic-like arginyl-tRNA synthetase to permit recognition by a position-20-independent mechanism. Bacillariophyta have undergone multiple endosymbiotic conversions with numerous horizontal gene transfers in all three genomic compartments (Armbrust et al. 2004; Bowler et al. 2008; Benoiston et al. 2017; Guillory et al. 2018). At which point the acquisition of tRNA^Arg^ genes coding for entities possessing C20 or U20 nucleotides took place remains a mystery. In modern-day organisms such characteristics in tRNA^Arg^ are rarely found outside Fungi and Microsporidia so that any concept regarding the origin of Diatoms would have to take the appearance of this facet of the genome into account.

Non-metazoans could be a source of other alternative mechanisms to match recognition elements (Online Resource 2). For example, within the Rhizaria, the phylum Endomyxa, with the data from only three species available, may provide, at least conceptually, another example of how recognition rules could be adapted to accommodate the available tRNAs. All three species have well-defined cytosolic as well as mitochondrial enzymes (Online Resource 1). However, the tRNA^Arg^ isoacceptors provided by the mitochondrial genome with A20 would require recognition by the cytosolic enzyme, whereas the nuclear tRNAs with U20 or C20 are destined for recognition by the mitochondrial enzyme. In this case differential targeting of the matching enzyme would provide a solution to the recognition problem. Unfortunately, in this case, because of the uncertainty of the N terminus derived from genomic data, no clear-cut targeting prediction could be obtained with the available algorithms.

The coevolution of aminoacyl-tRNA synthetases with their tRNA partners evidently relies not only on the structural plasticity of tRNA molecules (Giegé and Eriani 2021) but also on the adaptation of aminoacyl-tRNA synthetases to the binding of recognition domains present in ancestral tRNAs as has been pointed out for metazoan mitochondrial aminoacyl-tRNA synthetases (Neuenfeldt et al. 2013). This is in line with the hypothesis that emerging aminoacyl-tRNA synthetases adapted to an already established tRNA (De Pouplana et al. 1998) and is consistent with the prediction that tRNAs, as relics of the RNA world (Kühnlein et al. 2021), preceded their synthetases (Nagel and Doolittle 1995).

## Methods

For compiling the arginyl-tRNA synthetase collection, genomic (wgs) and transcriptome (tsa) NCBI and other databases were searched using TBLASTN. Accession numbers of the entries containing the corresponding arginyl-tRNA synthetase genes are given in Online Resource 3. Putative full length protein sequences were extracted from genomic hits manually by homology-based alignment FGENESH + (http://www.softberry.com/) by scanning for protein similarity using the corresponding or closely related organism-specific gene-finding parameters. For non-metazoans the available parameters are somewhat limited making N-terminal predictions, in particular, less reliable in the absence of transcriptome data. Multiple alignments were created with CLUSTALΩ (Madeira et al. 2019) and depicted in GENEDOC (Nicholas and Nicholas 1997). Sequences were classified as being of the cytosolic- or mitochondrial-type by visual inspection of the signature regions; GDYQ, KFKTR (for cytosolic) and 5∆MSTR (for mitochondrial) (Igloi 2020a). Organelle target prediction was performed on-line with TARGETP (Emanuelsson et al. 2000), DEEPLOC (Almagro Armenteros et al. 2017), MULOCDEEP (Jiang et al. 2021), HECTAR (Gschloessl et al. 2008) and BUSCA (Savojardo et al. 2018).

For tRNA sequences of non-metazoans neither the badly outdated tRNA database (Jühling et al. 2009) nor the limited genomic tRNA database (Chan and Lowe 2016) was found to be adequate. tRNAs for each organism were therefore recovered from annotated NCBI entries or by BLASTN followed by tRNAscan-SE (Chan et al. 2021). After MUSCLE alignment (Edgar 2004) of tRNA isoacceptors, sequences clustering with annotated mitochondrial or plastid tRNAs were re-examined. Plastid and mitochondrial tRNA isoacceptors form clusters so that one can identify potentially mis-annotated tRNAs. One such example is *Chaetoceros neogracilis* transcriptome HBTS01037129 which is identical to its annotated plastid genome (MW004650). Where no organelle genome data is available, this clustering approach is a tool to distinguish between organelle-encoded and mis-annotated nuclear-encoded tRNAs.

## Supplementary Information

Below is the link to the electronic supplementary material.

Supplementary file1 (PDF 573 KB)

Supplementary file2 (PDF 337 KB)

Supplementary file3 (PDF 350 KB)
