# Supplementary material for: Evolutionary Adjustment of tRNA Identity Rules in Bacillariophyta for Recognition by an Aminoacyl-tRNA Synthetase Adds a Facet to the Origin of Diatoms
Source: J Mol Evol. 2022 Mar 24;90(2):215–26. doi: 10.1007/s00239-022-10053-5 (PMC8975779; doi:10.1007/s00239-022-10053-5)
Supplement: Supplementary file 1 — Supplementary file1 (PDF 573 KB) [file 239_2022_10053_MOESM1_ESM.pdf]

Clade or Higher Classification	Phylum	Class/Order	No of Sequences mined	Species	Cyto ArgRS Type	Mito ArgRS Type	Cyto tRNA N20	Encoded Mito tRNA-Arg	Mito tRNA N20	Encoded Plastid tRNA-Arg	Plastid tRNA N20
**Amoebozoa**	**Discosea**		8	*Acanthamoeba lenticulata*		x	A	no	~		
				Balamuthia mandrillaris	x	x	A	no	~		
				Paramoeba pemaquidensis	x	x	U	yes	U		
				Stygamoeba regulata	x			?			
				Vermistella antarctica	x			?			
**Amoebozoa**	**Evosea**		22	*Acytostelium subglobosum*	x	x	A	yes	U		
				Cavenderia fasciculata	x	x	A	yes	U		
				Dictyostelium (Cavenderia) firmibasis	x	x	A	yes	U		
				Hagiwaraea rhizopodium		x		?			
				Heterostelium album	x	x	A	yes	U		
				Physarum polycephalum		x	A	no	~		
				Polysphondylium pallidum	x			?			
				Raperostelium potamoides		x	A	yes	C		
				Speleostelium caveatum	x	x	A	yes	U		
				Tieghemostelium lacteum	x	x	A	yes	U		
**Alveolata**	**Apicomplexa**[1]	**Aconoidasida**	34	*Babesia bigemina*	x	x?	A	no	~	yes	U
		**Conoidasina**		*Besnoitia besnoiti*	x		A	no	~	yes	A, U
		**Aconoidasida**		*Cardiosporidium cionae*	x		A	no	~	yes	A
		**Conoidasina**		*Cephaloidophora cf. communis*	x						
		**Conoidasina**		*Cyclospora cayetanensi1, Cyclospora cayetanensi2, Cyclospora cayetanensi3*	x	x?	A	no	~	yes	A
		**Conoidasina**		*Digyalum oweni*	x		A	no	~		
		**Conoidasina**		*Eimeria acervulina*	x		A	no	~	yes	A
		**Conoidasina**		*Eleutheroschizon duboscqi*	x		A	no	~	?	
		**Aconoidasida**		*Haemoproteus tartakovskyi*	x		A, C	no	~	yes	A

		**Conoidasina**		*Hammondia hammondi*	x		A	no	~	yes	U, A
		**Aconoidasida**		*Hepatocystis sp*	x		A	no	~	yes	A
		**Conoidasina**		*Neospora caninum*	x		A	no	~	yes	U, A
		**Aconoidasida**		*Nephromyces sp*	x		A	no	~	yes	A
		**Aconoidasida**		*Plasmodium fragile*	x		A	no	~	yes	A
		**Conoidasina**		*Polyrhabdina sp*	x	x?		no	~	?	
		**Conoidasina**		*Porospora cf. gigantea*	x			no	~	?	
		**Conoidasina**		*Rhytidocystis sp*	x		A	no	~	?	
		**Conoidasina**		*Sarcocystis neurona*	x			no	~	?	
		**Conoidasina**		*Siedleckia nematoides*	x	x	A	no	~	?	
		**Aconoidasida**		*Theileria parva*	x		A	no	~	yes	A, U[2]
		**Conoidasina**		*Toxoplasma gondii*	x		A	no	~	yes	U, A
**Alveolata**	**Ciliophora**[1]	**Heterotrichea**	15	*Condylostoma magnum*	x		A, U	no	~		
		**Litostomatea**		*Entodinium caudatum*	x		A	no	~		
		**Spirotrichea**		*Euplotes focardii*	x		A	no	~		
		**Oligohymenophorea**		*Ichthyophthirius multifiliis*	x		U	no	~		
		**Spirotrichea**		*Laurentiella sp*	x		A	no	~		
		**Spirotrichea**		*Oxytricha trifallax*	x		A	no	~		
		**Oligohymenophorea**		*Paramecium tetraurelia*	x		U	no	~		
		**Heterotrichea**		*Spirostomum semivirescens*	x			no	~		
		**Heterotrichea**		*Stentor coeruleus*	x		A	no	~		
		**Spirotrichea**		*Sterkiella histriomuscorum*	x		A	no	~		
		**Spirotrichea**		*Stylonychia lemnae*	x		A	no	~		
		**Spirotrichea**		*Tetmemena sp*	x		A	no	~		
		**Oligohymenophorea**		*Tetrahymena thermophila*	x		U	no	~		
		**Spirotrichea**		*Urostyla_sp*	x		A	no	~		
**Alveolata**	**Dinophyceae**[1]		31	*Alexandrium tamarense1, Alexandrium tamarense2*	x			no	~		
				Amphidinium carterae1, Amphidinium carterae2	x		A	no	~		
				Brandtodinium nutricula	x			no	~		
				Crypthecodinium cohnii	x			no	~		

				Gambierdiscus excentricus1, Gambierdiscus excentricus2	x			no	~		
				Gonyaulax spinifera	x			no	~		
				Gymnodinium catenatum1, Gymnodinium catenatum2, Gymnodinium catenatum3	x		A	no	~		
				Hematodinium sp	x			no	~		
				Heterocapsa triquetra	x			no	~		
				Karenia brevis	x			no	~		
				Karlodinium veneficum1, Karlodinium veneficum2	x		A	no	~		
				Lingulodinium polyedrum1, Lingulodinium polyedrum2	x		A	no	~		
				Noctiluca scintillans	x			no	~		
				Oxyrrhis marina	x		A	no	~	yes	A
				Pelagodinium beii	x			no	~		
				Polarella glacialis	x		A	no	~		
				Prorocentrum minimum1, Prorocentrum minimum2	x		A	no	~	yes	A
				Scrippsiella hangoei1, Scrippsiella hangoei2	x			no	~		
				Symbiodinium sp1, Symbiodinium sp2	x		A	no	~		
				Togula jolla	x			no	~		
**Alveolata**	**Perkinsozoa**		1	*Perkinsus marinus*	x		A	yes	A		
**Apusozoa**			2	*Thecamonas trahens*[3]	x		A, C, U	yes	A		
**Choanozoa**	**Choanoflagellata**	**Acanthoecida**	22	*Acanthoeca spectabilis*		x	A,C	?			
		**Craspedida**		*Choanoeca perplexa*	x		A	?			
		**Acanthoecida**		*Diaphanoeca grandis*		x	A	?			
		**Acanthoecida**		*Didymoeca costata*	x	x		?			
		**Craspedida**		*Hartaetosiga gracilis*	x	x	A, C	?			
		**Acanthoecida**		*Helgoeca nana*	x	x		?			
		**Craspedida**		*Microstomoeca roanoka*	x	x		?			
		**Craspedida**		*Mylnosiga fluctuans*	x	x	A	?			
		**Craspedida**		*Monosiga brevicollis*	x	x	A, U	yes	U		
		**Craspedida**		*Salpingoeca rosetta*	x	x	A, C	?			

		**Acanthoecida**		*Savillea parva*	x	x	A	?			
		**Acanthoecida**		*Stephanoeca diplocostata*	x	x	A	?			
**Cryptomonada**	**Cryptophyta**		10	*Chroomonas sp1, Chroomonas sp2*	x		A	yes	A	yes	A
				Cryptomonas paramecium	x		A	yes	A	yes	A
				Goniomonas pacifica	x		A	yes	A	yes	A
				Guillardia theta1, Guillardia theta2	x		A	yes	A	yes	A
				Hanusia phi	x			?			
				Hemiselmis rufescens1, Hemiselmis rufescens2	x		A	yes	A	yes	A
				Rhodomonas abbreviata	x		A	yes	A	yes	A
**Discoba**	**Euglenophyta**		2	*Euglena gracilis1, Euglena gracilis2*	x		A	yes	A		
**Discoba**	**Euglenozoa**	**Kinetoplastida**[1]	15	*Angomonas deanei*	x		A	no	~		
				Crithidia fasciculata	x		A	no	~		
				Endotrypanum monterogeii	x		A	no	~		
				Herpetomonas muscarum	x		A	no	~		
				Leishmania mexicana	x		A	no	~		
				Leptomonas pyrrhocoris	x		A	no	~		
				Lotmaria passim	x		A	no	~		
				Neobodo designis	x			no	~		
				Perkinsela sp.	x		U	no	~		
				Phytomonas serpens	x		A	no	~		
				Porcisia hertigi	x		A	no	~		
				Strigomonas galati	x		A	no	~		
				Trypanoplasma borreli	x			no	~		
				Trypanosoma cruzi	x		A	no	~		
				Vickermania ingenoplastis	x			no	~		
**Discoba**	**Percolozoa**	**Heterolobosea**	12	*Acrasis kona*				no	~		
				Naegleria fowleri	x	x	A	yes	U		
				Neovahlkampfia damariscottae	x	x	C, U	?			
				Percolomonas cosmopolitus1, Percolomonas cosmopolitus2, Percolomonas cosmopolitus3		x		?			

				Pharyngomonas kirbyi	x			yes	A		
				Willaertia magna	x	x	A	yes	U[4]		
**Fungi**	**Cryptomycota**		2	*Paramicrosporidium saccamoebae*		x	A	yes	U, A		
				Rozella allomycis		x	A	no	~		
**None**	**Haptista**	**Haptophyta**	20	*Calcidiscus leptoporus*	x			?			
				Chrysochromulina spCCMP291 1, Chrysochromulina spCCMP291 2	x		A	yes	A	yes	A
				Chrysotila carterae1, Chrysotila carterae2	x			?			
				Coccolithus braarudii1, Coccolithus braarudii2	x			?			
				Diacronema lutheri	x			yes	A		
				Emiliania huxleyi 1, Emiliania huxleyi 2	x		A	yes	A	yes	A
				Exanthemachrysis gayraliae	x			?			
				Gephyrocapsa oceanica	x		A	yes	A		
				Isochrysis galbana1, Isochrysis galbana2	x		A	yes	A		
				Phaeocystis antarctica1, Phaeocystis antarctica2	x		A	yes	A	yes	A
				Prymnesium parvum1, Prymnesium parvum2	x		A	yes	A		
				Scyphosphaera apsteinii1, Scyphosphaera apsteinii2	x			?			
**None**	**Rhodophyta**		15	*Agarophyton vermiculophyllum*	x		A	yes	A		
				Asparagopsis taxiformis	x		A, C[5]	yes	A		
				Betaphycus philippinensis	x		A	?			
				Chondrus crispus	x		A	yes	A	yes	A
				Cyanidioschyzon merolae	x			?			
				Dumontia simplex	x			?			
				Eucheuma denticulatum	x			yes	A		
				Galdieria sulphuraria	x		A	no	~	yes	A
				Gracilaria chilensis	x			yes	A		
				Gracilariopsis lemaneiformis	x		A	yes	A		
				Kappaphycus alvarezii	x		A	yes	A		
				Laurencia pacifica	x		A	?			
				Neopyropia yezoensis	x		A	yes	A		

				Porphyra umbilicalis	x			yes	A		
				Porphyridium purpureum	x		A	?		yes	A
**Opalozoa**		**Bicosoecida**	3	*Bicosoecida sp*	x			?			
				Cafeteria roenbergensis	x			yes	A		
				Halocafeteria seosinensis	x		A	?			
**Opisthokonta**	**Filasterea**		4	*Capsaspora owczarzaki*	x	x	U	yes	U, C		
				Filasterea sp	x	x		?			
**Opisthokonta**		**Ichthyosporea**	10	*Amoebidium parasiticum*	x	x	A	?			
				Creolimax fragrantissima	x	x	A, U	?			
				Ichthyophonus hoferi	x	x	A	?			
				Ichthyosporea sp	x	x	A, C	?			
				Sphaeroforma arctica	x	x	A, U	yes	A, C		
**Opisthokonta**		**Rotosphaerida**	1	*Fonticula alba*		x	A	yes	A		
**Rhizaria**	**Cercozoa**	**Chlorarachniophyceae**	7	*Amorphochlora amoebiformis*		x	A	?			
				Bigelowiella natens	x	x	A	no	~	yes	A
				Chlorarachnion reptans		x		?			
				Lotharella globosa	x	x		?		yes	A
				Partenskyella glossopodia	x	x		?		yes	A
**Rhizaria**	**Endomyxa**	**Phytomyxea**[6]	6	*Plasmodiophora brassicae*	**x**	x	U, C	yes	A	~	~
				Polymyxa betae	x	x	U	yes	A		
				Spongospora subterranea	x	x	U	yes	A		
**Rhizaria**	**Imbricatea**		1	*Paulinella chromatophora*	x		A	no	~	yes	A
**Rhizaria**	**Foraminifera**		2	*Globobulimina GloT15*		x	A, U	?			
				Reticulomyxa filosa		x	A	?			
**Rhizaria**	**Unclassified**		1	*Rhizaria sp*		x	U	?			
**Stramenopiles**	**Bacillariophyta**		51	*Asterionella formosa 1, Asterionella formosa 2*	x		A, C, U	yes	A	yes	A
				Asterionellopsis glacialis1, Asterionellopsis glacialis2	x		C, U	yes	A	yes	A
				Chaetoceros neogracilis1, Chaetoceros neogracilis2	x		A, U	yes	A	yes	A
				Conticribra weissflogii1, Conticribra weissflogii2	x		C	yes	A	yes	A

			Corethron pennatum1, Corethron pennatum2	x		?		?	
			Coscinodiscus wailesii1, Coscinodiscus wailesii2	x	U	yes	A	yes	A
			Cylindrotheca closterium1, Cylindrotheca closterium2	x	U	yes	A	yes	A
			Dactyliosolen fragilissimus1, Dactyliosolen fragilissimus2	x		?		?	
			Ditylum brightwellii1, Ditylum brightwellii2	x	C, U	?		?	
			Fistulifera solaris1, Fistulifera solaris2	x	C, U	yes	A	yes	A
			Fragilariopsis cylindrus 1, Fragilariopsis cylindrus 2	x	C, U	yes	A	yes	A
			Guinardia flaccida1, Guinardia flaccida2	x		yes	A	yes	A
			Leptocylindrus danicus	x		yes	A	yes	A
			Minidiscus sp	x		?		?	
			Minutocellus polymorphus1, Minutocellus polymorphus2	x		?		?	
			Navicula sp1, Navicula sp2	x	C	yes	A	?	
			Nitzschia sp1, Nitzschia sp2	x	U	yes	A	yes	A
			Odontella aurita	x	?	yes	A	yes	A
			Phaeodactylum tricornutum 1, Phaeodactylum tricornutum 2	x	U	yes	A	yes	A
			Pseudo-nitzschia multistriata, Pseudo-nitzschia fraudulenta	x	U	yes	A	yes	A
			Skeletonema marinoi1, Skeletonema marinoi2	x	U	yes	A	?	
			Stephanopyxis turris1, Stephanopyxis turris2	x		?		?	
			Synedra sp1, Synedra sp2	x		?		?	
			Synedropsis cf. recta	x		?		?	
			Thalassiosira pseudonana 1, Thalassiosira pseudonana 2	x	C, U	yes	A	yes	A
			Tryblionella compressa1, Tryblionella compressa2	x		?		?	
**Stramenopiles**	**Eustigmatophyceae**	2	*Nannochloropsis gaditana1, Nannochloropsis gaditana2*	x	A	yes	A	yes	A
**Stramenopiles**	**Labyrinthulomycota**	8	*Aplanochytrium kerguelense 1, Aplanochytrium kerguelense 2*	x		?			
			Aurantiochytrium sp	x	A, U	no	~		
			Schizochytrium sp	x	A, U	no	~		
			Hondaea fermentalgiana	x	C, U	?			

				Labyrinthula sp	x			yes[7]	A		
				Thraustochytrium sp1, Thraustochytrium sp2	x		A, C	no	~		
**Stramenopiles**	**Oomycota**		42	*Achlya hypogyna*	x		A	yes	A, U		
				Albugo candida1, Albugo candida2	x	x	A	?			
				Aphanomyces invadans 1, Aphanomyces invadans 2	x	x	A, C[8]	yes	A, U		
				Bremia lactucae1, Bremia lactucae2	x	x	A	yes	A, U		
				Globisporangium iwayamae1, Globisporangium iwayamae2	x	x	A	yes	A, U		
				Hyaloperonospora arabidopsidis 1, Hyaloperonospora arabidopsidis 2	x	x	A	?			
				Lagenidium giganteum	x		A	?			
				Paralagenidium karlingii1, Paralagenidium karlingii2	x	x	A	?			
				Peronospora effusa		x	A	yes	A, U		
				Phytophthora kernoviae1, Phytophthora kernoviae2, Phytophthora kernoviae3	x	x	A	yes	A, U		
				Phytopythium vexans1, Phytopythium vexans2		x	A	?			
				Pilasporangium apinafurcum 1, Pilasporangium apinafurcum 2		x	A	?			
				Plasmopara halstedii1, Plasmopara halstedii2		x	A, U	yes	A, U		
				Pseudoperonospora cubensis		x	A	yes	A, U		
				Pythium insidiosum 1, Pythium insidiosum 2		x	A	yes	A, U		
				Saprolegnia parasitica 1, Saprolegnia parasitica 2		x	A	yes	A, U		
				Sclerospora graminicola 1, Sclerospora graminicola 2		x	A	?			
				Thraustotheca clavata	x		A	yes	A, U		
**Straminopiles**		**Opalinata**	1	*Blastocystis hominis*	x		A	no	~		
**Stramenopiles**		**Pelagophycea**	2	*Aureococcus anophagefferens1, Aureococcus anophagefferens2*	x		A	yes	A	yes	A
**Stramenopiles**		**Phaeophyceae**	35	*Agarum clathratum*	x			?		?	
				Alaria esculenta	x			?		?	
				Chorda filum	x			?		?	
				Cladosiphon okamuranus 1,Cladosiphon okamuranus 2, Cladosiphon okamuranus 3	x		A	yes	A	yes	A
				Colpomenia sinuosa	x			yes	A	?	

				Costaria costata	x		yes	A	yes	A
				Cymathaere triplicata	x		?		?	
				Desmarestia viridis1, Desmarestia viridis2	x		yes	A	?	
				Dictyopteris undulata	x		yes	A	yes	A
				Ecklonia radiata	x		?		?	
				Ectocarpus siliculosus 1, Ectocarpus siliculosus 2	x	A	yes	A	yes	A
				Egregia menziesii	x		?		?	
				Fucus ceranoides	x	A	yes	A	yes	A
				Ishige okamurae	x		?		?	
				Laminaria digitata	x	A	yes	A	yes	A
				Lessonia nigrescens1, Lessonia nigrescens 2	x		yes	A	yes	A
				Macrocystis pyrifera	x	A	yes	A	yes	A
				Nereocystis luetkeana 1, Nereocystis luetkeana 2	x		yes	A	?	
				Petalonia fascia	x		yes	A	?	
				Pleurophycus gardneri	x		?		?	
				Pseudochorda nagaii1, Pseudochorda nagaii2	x		?		?	
				Pterygophora californica	x		?		?	
				Punctaria latifolia	x		?		?	
				Saccharina japonica 1, Saccharina japonica 2	x	A	yes	A	yes	A
				Sargassum integerrimum	x		yes	A	yes	A
				Scytosiphon dotyi	x		yes	A	yes	A
				Undaria pinnatifida1, Undaria pinnatifida2	x	A	?		?	
**Straminopiles**		**Xanthophyceae**	3	*Heterococcus sp*	x	A	?		?	
				Tribonema minus	x	A	?		?	
				Vaucheria litorea	x		?		yes	A
**Viridiplantae**	**Chlorophyta**		16	*Asterochloris sp*	x	A	yes		yes	
				Auxenochlorella protothecoides	x		?		?	
				Bathycoccus prasinos	x	A	yes	A	yes	A
				Botryococcus braunii	x	A	yes	A	yes	A

	Chlamydomonas reinhardtii	x	A	no	~	yes	A
	Chlorella pyrenoidosa	x	A	yes	A	yes	A
	Coccomyxa subellipsoidea	x	A	yes	A	yes	A
	<u>*Dunaliella primolecta*</u>	x		?		?	
	Helicosporidium sp	x		yes	A, (U?)	?	
	Klebsormidium flaccidum	x	A	yes	A	yes	A
	Micromonas pusilla	x	A	yes	A	yes	A
	Ostreococcus tauri	x	A	yes	A	yes	A
	Picochlorum sp	x	A	yes	A	yes	A
	Tetraselmis (Platymonas) sp	x	A	no	~	?	
	Ulva lactuca	x	A	yes	A	yes	A
	Volvox carteri	x	A	no	~	yes	A

Online Resource 1
Compilation of non-metazoan arginyl-tRNA synthetases according to their taxonomic positions and their classification into cytosolic or mitochondrial forms (x). Where possible, the nature of the base at position 20 in the corresponding cytosolic, mitochondrial and plastid tRNAs is given. Species for which no tRNA data are available are underlined. Uncertainties are denoted by "?". For species whose mitochondrion does not encode $tRNA^{Arg}$, the entry for position 20 is "~". Accession numbers of data base entries from which the genes were derived are listed in Online Resource 3. ArgRS; arginyl-tRNA synthetase

[1] Species within divisions for which no mitochondrial-encoded $tRNA^{Arg}$ are known, to date, have been assumed to lack such tRNAs, even when no mitochondrial genome is available
[2] *T.parva* has A20 in both apicoplast tRNAs; *T.equi* has U20 in both but with some non-canonical structures
[3] Indistinct FGDYQ sequence
[4] DNA segment from wgs database containing this $tRNA^{Arg}$ also harbours 24 other tRNAs but is not annotated as being mitochondrial
[5] Is the only C20-containing $tRNA^{Arg}$ in the Rhodophyte wgs database
[6] Derived from genomic sequences only. Appear to have different gene fragmentation pattern for each species
[7] from Labyrinthulomycetes sp. (unclassified) mitochondrial genome
[8] A pair of encoded $tRNA_{TCT}$ isoacceptors with C20 and A20 nucleotides

Evolutionary adjustment of tRNA identity rules in Bacillariophyta for recognition by an aminoacyl-tRNA synthetase adds a facet to the origin of Diatoms
J.Mol.Evol.
Gabor L. Igloi, University of Freiburg; igloi@biologie.uni-freiburg.de
Online Resource 1
