# Supplementary material for: Evolutionary Adjustment of tRNA Identity Rules in Bacillariophyta for Recognition by an Aminoacyl-tRNA Synthetase Adds a Facet to the Origin of Diatoms
Source: J Mol Evol. 2022 Mar 24;90(2):215–26. doi: 10.1007/s00239-022-10053-5 (PMC8975779; doi:10.1007/s00239-022-10053-5)
Supplement: Supplementary file 2 — Supplementary file2 (PDF 337 KB) [file 239_2022_10053_MOESM2_ESM.pdf]

Non-Metazoan Arginyl-tRNA Synthetase Classification and tRNA Recognition of Organisms with Questionable Identity Rules

Clade	Phylum	Class/Order	Species	Cyto Arginyl tRNA synthetase Type	Mito Arginyl-tRNA synthetase Type	Cyto tRNA N20	Encoded Mito tRNA-Arg	Mito tRNA N20	Encoded Plastid tRNA-Arg	Plastid tRNA N20	Verdict on likely identity element erosion (see text)
**Amoebozoa**	**Discosea**		*Paramoeba pemaquidensis*	x	x	U	yes	U			Questionable
**Alveolata**	**Apicomplexa**		*Haemoproteus tartakovskyi*	x	x	A, C	no	~	yes	A	Uncertain
	**Ciliophora**		*Condylostoma magnum*	x		A, U	no	~			
			Ichthyophthirius multifiliis	x		U	no	~			Potential loss of A20 requirement by cytosolic enzyme
			Paramecium tetraurelia	x		U	no	~			
			Tetrahymena thermophila	x		U	no	~			
**Apusozoa**			*Thecamonas trahens*	x		A, C, U	yes	A			Possible but insufficient data.
**Choanozoa**	**Choanoflagellata**		*Hartaetosiga gracilis*	x	x	A, C	?	?			
			Monosiga brevicollis	x	x	A, U	yes	U			Uncertain
			Salpingoeca rosetta	x	x	A, C	?	?			
**Discoba**	**Euglenozoa**	**Kinetoplastida**	*Perkinsela sp.*	x		U	no	~			Possible
	**Percolozoa**	**Heterolobosea**	*Neovahlkampfia damariscottae*	x	x	C, U	?	?			Possible
**Opisthokonta**	**Filasterea**		*Capsaspora owczarzaki*	x	x	U	yes	U, C			Possible
		**Ichthyosporea**	*Creolimax fragrantissima*	x	x	A, U	?	?			
			Ichthyosporea sp	x	x	A, C	?	?			Possible
			Sphaeroforma arctica	x	x	A, U	yes	A, C			
**Rhizaria**	**Endomyxa**	**Phytomyxea**	*Plasmodiophora brassicae*	**x**	x	U, C	yes	A			No identity change required if appropriately targeted
			Polymyxa betae	x	x	U	yes	A			
			Spongospora subterranea	x	x	U	yes	A			
**Stramenopiles**		**Labyrinthulomycota**	*Aurantiochytrium sp*	x		A, U	no	~			
			Schizochytrium sp	x		A, U	no	~			Possible
			Hondaea fermentalgiana	x		C, U	no	~			
			Thraustochytrium sp	x		A, C	no	~			

Amoebozoa

30 Sequences from 15 species are divided between two phyla. N termini are highly divergent and prediction with FGENESH+ (http://www.softb erry.com/) appears unreliable. In the case of Balamuthia cytosolic arginyl-tRNA synthetase, for example, the 5’ end of its CDS may lie in a stretch of unresolved N nucleotides, accounting for the extremely truncated N terminus.

Amoebozoa possess both cytosolic and mitochondrial forms of the enzyme. The mitochondrial arginyl-tRNA synthetases are characterised by a typical 5△MSTR deletion (Igloi 2020). In these organisms, mitochondrial $tRNA^{Arg}{}_{UCU}$, when encoded by the organelle genome, has U20 which would not be recognized by a cytosolic enzyme. In contrast, the cytosolic $tRNA^{Arg}{}_{UCU}$ and $tRNA^{Arg}{}_{ACG}$ have A20, the essential identity element for the cytosolic form of the enzyme.

Raperostelium and Speleostelium have no mitochondrial tRNA sequence in the database but “genomic” transcriptome annotation has segments with BLASTN similarity to other Amoebozoa mitochondrial $tRNA^{Arg}$ (with C20 or U20) (Igloi 2019) and a gene order within a tRNA gene cluster that is very similar to *D.discoideum* mitochondrial genome.

Paramoeba pemaquidensis cytosolic arginyl-tRNA synthetase forms an exception both in its sequence, having a very poorly defined GDYQ motif (Igloi 2019). Consequently, all its cytosolic $tRNA^{Arg}$ isoacceptors have U20. Nevertheless, one should recall that Paramoeba is associated with the kinetoplastid endosymbiont Perkinsela (Tanifuji et al. 2017), whose $tRNA^{Arg}$ isoacceptors also possess U20. Moreover, the source of the genomic material was specified as being “enriched in endosymbiont nuclear DNA but also contained host nuclear DNA and mitochondrial DNA from both organisms” (Tanifuji et al. 2017).The origin of the arginyl-tRNA synthetase is, therefore, uncertain. Furthermore, lateral gene transfer between host and symbiont cannot be excluded (Cenci et al. 2016). Hence some caution in classifying the Paramoeba arginyl-tRNA synthetase as an exception to canonical recognition rules might be exercised.

Apicomplexa

At least two genes can be detected in 9 of 21 species, of which one clearly deviates from the canonical cytosolic form in having sequence motifs that diverge widely from the typical GDYQ domain. The potential of apicomplexan DNA contaminating animal genome and transcriptome assemblies has been noted (Borner and Burmester 2017). In the same way metazoan contamination of environmental Apicomplexan samples might be anticipated. In this regard one needs to be aware that a *Cyclospora cayetanensis* arginyl-tRNA synthetase gene product (Acc.No. PDMO01000129) has a close resemblance (70% sequence identity) to metazoan mitochondrial arginyl-tRNA synthetase and *Siedleckia nematoides* (Acc. No. GHVV01320568) as well as *Polyrhabdina sp.* (Acc.No. GHVP01022720) have the typical metazoan mitochondrial 5△MSTR motif.

The mitochondrial genome does not encode $tRNA^{Arg}$ isoacceptors which need to be imported from the cytosol (Rubio and Hopper 2011). Nuclear encoded $tRNA^{Arg}$ isoacceptors consistently have A20 (*H. tartakovskyi* appears to be an exception with $tRNA^{Arg}{}_{UCG}$ possessing C20 (Acc.No. LSRZ01001064)), but it is the only divergence from A20 in all 42 despite them being derived from Rhodophyta (Striepen 2011) whose modern-day $tRNA^{Arg}$ isoacceptors all carry A20. The uncertainty is enhanced by the fact that, *Theileria parva* has A20 in both apicoplast-encoded $tRNA^{Arg}$ isoacceptors whereas *T.equi* has U20 in the same isoacceptors (but with different sequence). Targeting predictions are inconclusive.

Apusozoa

Genomic data from only one species exists. This reveals the presence of two enzymes. The GDYQ motif is indiscernible in in both. The cytosolic enzyme needs to recognize A,C,U at position 20 with two $tRNA^{Arg}_{UCG}$ isodecoders being characterised by both A20 and U20 (Acc.No. GL349443, GL349464).

Choanozoa:

Both cytosolic and mitochondrial enzyme types have been extracted from the databases for most of the 12 species. However, tRNA data are only available in one instance and confirms the need for an A20-insensitive mitochondrial enzyme. The mitochondrial sequences are characterized by the missing “GDYQ-like” domain and the typical “5△MSTR-like” feature. However, the nuclear genome of several species encode tRNAs with U20 or C20 as well as A20. It is not clear, whether the “mitochondrial” enzyme is also retained in the cytosolic to arginylate the non-A20 isoacceptors.

Ciliophora

Only one type of arginyl-tRNA synthetase is apparent and most species are represented by genomic data only, resulting in apparently atypical insertions in the derived translations. The GDYQ-motif poorly retained. Nevertheless, the enzymes are classified as being of the cytosolic type, in view of the intact KFKTR region. No mitochondrial-encoded $tRNA^{Arg}$ are known (Gagat et al. 2017) and need to be imported. The cytosolic $tRNA^{Arg}$ possess A20, except for the three organisms of Class Oligohymenophorea which consistently have U20 (Igloi 2019).

Kinetoplastida

All 15 species are restricted to having a single cytosolic-like arginyl-tRNA synthetase with typical GDYQ and KFKTR motifs. However, Perkinsela, an endosymbiont of Paramoeba (see above) and noted for its significant level of divergence from other kinetoplastids (Dyková et al. 2003) has variations in other regions for recognizing U20.

Percolozoa

The five species (four genera) providing BLAST hits, all have both cytosolic arginyl-tRNA synthetases with KFKTR and mitochondrial enzymes with no GDYQ but with MSSR motifs. Only Neovahlkampfia encodes a nuclear tRNA with C20, U20 and its cytosolic enzyme reveals a corrupt GDYQ sequence having replaced the Q by E.

Filasterea

With the availability of only two organisms, generalisations are not possible. Both species possess cytosolic and mitochondrial enzymes although in the case of *Capsaspora owczarzaki* the GDYQ motif is nor recognizable in the cytosolic form and neither nuclear nor mitochondrial $tRNA^{Arg}$ have A20. No tRNA data is available for *Filasterea sp.*

Ichthyosporea

Cytosolic and mitochondrial arginyl-tRNA synthetases have been identified for all five species. According to the classification rules, the mitochondrial enzymes lack the GDYQ domain and reveal the 5△MSTR feature. The mitochondrial $tRNA^{Arg}$ isoacceptors have A20, U20, and C20. But U20 and C20 are also found in cytosolic tRNAs with no obvious deviation from the canonical sequence of the cytosolic enzymes that would explain the loss of A20 sensitivity.

Labyrinthulomycota

Labyrinthulomycota have exceptionally long N-terminal extensions leading to some of the longest proteins of this class of enzymes with almost 800 amino acids. There may be two forms per species but both are characteristically of cytosolic origin although the GDYQ motif is atypical (frequently, PDFQ). The G to P replacement and concomitant reduction in the chain flexibility may explain the altered D-loop interaction, permitting the recognition of the C20 or U20 in the cytosolic $tRNA^{Arg}$. These are then also imported into the mitochondria.

References

Bornertrna J, Burmester T (2017) Parasite infection of public databases: A data mining approach to identify apicomplexan contaminations in animal genome and transcriptome assemblies. BMC Genomics 18:100. doi: 10.1186/s12864-017-3504-1

Cenci U, Moog D, Curtis BA, et al (2016) Heme pathway evolution in kinetoplastid protists. BMC Evol Biol 16:109. doi: 10.1186/s12862-016-0664-6

Dyková I, Fiala I, Lom J, Lukeš J (2003) Perkinsiella amoebae-like endosymbionts of Neoparamoeba spp., relatives of the kinetoplastid Ichthyobodo. Eur J Protistol 39:37–52. doi: 10.1078/0932-4739-00901

Gagat P, Mackiewicz D, Mackiewicz P (2017) Peculiarities within peculiarities – dinoflagellates and their mitochondrial genomes. Mitochondrial DNA Part B 2:191–195. doi: 10.1080/23802359.2017.1307699

Igloi GL (2020) Molecular evidence for the evolution of the eukaryotic mitochondrial arginyl-tRNA synthetase from the prokaryotic suborder Cystobacterineae. FEBS Lett 594:951–957. doi: 10.1002/1873-3468.13665

Igloi GL (2022) Compilation and Alignment of Eukaryotic Arginyl-tRNA Synthetases. In: Mendeley Data. https://data.mendeley.com/datasets/ts4jbw9nft/3

Rubio MAT, Hopper AK (2011) Transfer RNA travels from the cytoplasm to organelles. Wiley Interdiscip Rev RNA 2:802–817. doi: 10.1002/wrna.93

Striepen B (2011) The apicoplast: a red alga in human parasites. Essays Biochem 51:111–125. doi: 10.1042/BSE0510111

Tanifuji G, Cenci U, Moog D, et al (2017) Genome sequencing reveals metabolic and cellular interdependence in an amoeba-kinetoplastid symbiosis. Sci Rep 7:11688. doi: 10.1038/s41598-017-11866-x

Evolutionary adjustment of tRNA identity rules in Bacillariophyta for recognition by an aminoacyl-tRNA synthetase adds a facet to the origin of Diatoms
J.Mol.Evol.
Gabor L. Igloi, University of Freiburg; igloi@biologie.uni-freiburg.de
Online Resource 2
