# Supplementary material for: Evolutionary Adjustment of tRNA Identity Rules in Bacillariophyta for Recognition by an Aminoacyl-tRNA Synthetase Adds a Facet to the Origin of Diatoms
Source: J Mol Evol. 2022 Mar 24;90(2):215–26. doi: 10.1007/s00239-022-10053-5 (PMC8975779; doi:10.1007/s00239-022-10053-5)
Supplement: Supplementary file 3 — Supplementary file3 (PDF 350 KB) [file 239_2022_10053_MOESM3_ESM.pdf]

**Species**	**Cyto ArgRS Type**		**Mito ArgRS Type**	
		**Accession Number**		
	**Genomic**	**TSA**	**Genomic**	**TSA**
Acanthamoeba lenticulata			CDFG01078352	IACY01007344
Balamuthia mandrillaris	LEOU01000743		LEOU01014529	
Paramoeba pemaquidensis		GEWA01004053		GEWA01007025
Stygamoeba regulata		HBLF01015726		
Vermistella antarctica		GELU01004528		
Acytostelium subglobosum	BAUZ01000018		BAUZ01000149	
Cavenderia fasciculata	ADHC01000003	GIOM01038025	GIOM01014483	
Dictyostelium (Cavenderia) firmibasis	AJWH01000365		AJWH01006818	GIQF01040785
Hagiwaraea rhizopodium		GIOY01010642	GIOY01011420	
Heterostelium album	ADBJ01000020		GIPA01003566	
Physarum polycephalum				GDRG01005745
Polysphondylium pallidum	ADBJ01000020	ADBJ01000038		
Raperostelium potamoides		GIOX01033976	GIOO01003964	
Speleostelium caveatum	RCTL01001915		RCTL01001917	
Tieghemostelium lacteum	LODT01000035	GIPF01015448	LODT01000004	GIPF01046888
Babesia bigemina	NC_027217		CCBM010000004	
Besnoitia besnoiti	NWUJ01000011			
Cardiosporidium cionae	JADAQX010000019, (JADAQX010001238+JADAQX010001134)	GIVE01001602		
Cephaloidophora cf. communis		GHVH01001002		
Cyclospora cayetanensi1, Cyclospora cayetanensi2, Cyclospora cayetanensi3	NW_019211145, PDMO01000013,		PDMO01000129[1]	
Digyalum oweni		GHVL01038146		
Eimeria acervulina	NW_013549849			
Eleutheroschizon duboscqi		GHVT01065224		
Haemoproteus tartakovskyi	LSRZ01000507	GGWD01005695		
Hammondia hammondi	NW_008634137			
Hepatocystis sp	CABPSV020001060			
Neospora caninum	NC_018389			
Nephromyces sp	JADHZB010000501, JADHZB010000519	GHIL01106088, GHIL01181227, GHIL01171613		
Plasmodium fragile	NW_012192614	XM_012480759		
Polyrhabdina sp		GHVP01050568		GHVP01022720
Porospora cf. gigantea	JAHYSE010000361			
Rhytidocystis sp		GHVS01059240		
Sarcocystis neurona	JAQE01000242			
Siedleckia nematoides		GHVV01321112		GHVV01320568
Theileria parva	AAGK01000001, AAGK01000004			
Toxoplasma gondii	LN714493			

Condylostoma magnum	CVLX01009788			
Entodinium caudatum		GHEK01027902		
Euplotes focardii	MJUV01068700	HBJK01033661		
Ichthyophthirius multifiliis	AEDN01001713			
Laurentiella sp	LASS02004151			
Oxytricha trifallax	AMCR01019535			
Paramecium tetraurelia	NW_001798971	FX880623		
Spirostomum semivirescens		GGNT01005402		
Stentor coeruleus	MPUH01000874			
Sterkiella histriomuscorum	LAST02003617			
Stylonychia lemnae	ADNZ03007317			
Tetmemena sp	LASU02018853			
Tetrahymena thermophila	NW_002476185			
Urostyla_sp	LASQ02005391			
Alexandrium tamarense1, Alexandrium tamarense2		GAIT01043575, GAIQ01046835		
Amphidinium carterae1, Amphidinium carterae2		HBNO01039160, HBNO01041335		
Brandtodinium nutricula		HBGW01088756		
Crypthecodinium cohnii		GFIV01041281		
Gambierdiscus excentricus1, Gambierdiscus excentricus2		GETL01012269, GETL01008749		
Gonyaulax spinifera		HBNG01064722		
Gymnodinium catenatum1, Gymnodinium catenatum2, Gymnodinium catenatum3		HBLW01016977, HBLW01029067, HBLW01055951		
Hematodinium sp		GEMP01020636		
Heterocapsa triquetra		HBLK01061713		
Karenia brevis		GFLM01039145		
Karlodinium veneficum1, Karlodinium veneficum2		HBOS01069635, GGWG01040322		
Lingulodinium polyedrum1, Lingulodinium polyedrum2		GABP01099160, GABP01011985		
Noctiluca scintillans		GELK01059782		
Oxyrrhis marina		HBQX01016561		
Pelagodinium beii		HBNF01090594		
Polarella glacialis		HBLC01058194		
Prorocentrum minimum1, Prorocentrum minimum2	JXLM01000238, JXLM01002807			
Scrippsiella hangoei1, Scrippsiella hangoei2		HBPM01009007, HBPM01091780		
Symbiodinium sp1, Symbiodinium sp2	GAFP01017137, GAKY01057484			
Togula jolla		HBKY01052867		
Perkinsus marinus	GG682469			
Thecamonas trahens	NW_013657645, ADVD01000720			

Acanthoeca spectabilis				GGPA01027531
Choanoeca perplexa		GGOP01020943		
Diaphanoeca grandis				GGPB01039897
Didymoeca costata		GGOQ01017063		GGOQ01009709
Hartaetosiga gracilis		GGOU01004703		GGOO01001903
Helgoeca nana		GGOR01004388		GGOR01023222
Microstomoeca roanoka		GGON01015976		GGON01009098
Mylnosiga fluctuans		GGOI01011751		GGOI01024155
Monosiga brevicollis	NW_001865091		NW_001865040	
Salpingoeca rosetta		GGOW01001636	NW_004754916	
Savillea parva		GGOL01002987		GGOL01000388
Stephanoeca diplocostata		GGOM01043382		GGOM01024840
Chroomonas sp1, Chroomonas sp2		ICPR01055017, ICPR01031141		
Cryptomonas paramecium		HBKS01043673		
Goniomonas pacifica		HBOP01054658		
Guillardia theta1, Guillardia theta2	NW_005434658, NW_005434651	HBKN01006356		
Hanusia phi		HBEO01018439		
Hemiselmis rufescens1, Hemiselmis rufescens2		HBMS01047417, HBMS01047465		
Rhodomonas abbreviata		HBKF01042791		
Euglena gracilis1, Euglena gracilis2	GDJR01076325, GDJR01084101			
Angomonas deanei	LXWQ01000789			
Crithidia fasciculata	AODS02000457			
Endotrypanum monterogeii	AOFS02000844			
Herpetomonas muscarum	AUXJ01007476			
Leishmania mexicana	NC_018331			
Leptomonas pyrrhocoris	NW_015438363			
Lotmaria passim	AHIJ01000160			
Neobodo designis		HBGF01001112		
Perkinsela sp.	LFNC01000110			
Phytomonas serpens	AIHY01001624			
Porcisia hertigi	JAFJZO010000027			
Strigomonas galati	AUXN01000620			
Trypanoplasma borreli		GHOB01010719		
Trypanosoma cruzi	NW_001849461			
Vickermania ingenoplastis	VFSE01000224			
Acrasis kona				
Naegleria fowleri	AWXF01000149		VFQX01000013	
Neovahlkampfia damariscottae	JABLTG010000021		JABLTG010000022	
Percolomonas cosmopolitus1, Percolomonas cosmopolitus2, Percolomonas cosmopolitus3				HBGD01001471, HBLE01012886,HBLE01009481

Pharyngomonas kirbyi		GECH01013841		
Willaertia magna	CADWAF010002650		CADWAF010001843	
Paramicrosporidium saccamoebae			MTSL01000174	
Rozella allomycis			ATJD01000982	
Calcidiscus leptoporus		HBER01047951		
Chrysochromulina spCCMP291 1, Chrysochromulina spCCMP291 2	JWZX01002193, JWZX01001859			
Chrysotila carterae1, Chrysotila carterae2		HBIZ01047847, HBIZ01040757		
Coccolithus braarudii1, Coccolithus braarudii2		HBEY01010025, HBEY01000181		
Diacronema lutheri		HBEB01013983		
Emiliania huxleyi 1, Emiliania huxleyi 2	AHAL01005489, AHAL01006405	HBIR01016636		
Exanthemachrysis gayraliae		HBNB01003555		
Gephyrocapsa oceanica		HBON01054623		
Isochrysis galbana1, Isochrysis galbana2		HBOM01021611, HBOM01021613		
Phaeocystis antarctica1, Phaeocystis antarctica2		GFUQ01117993, HBQY01009167		
Prymnesium parvum1, Prymnesium parvum2		HBJC01047810, HBJC01047809		
Scyphosphaera apsteinii1, Scyphosphaera apsteinii2		HBMI01007711, HBMI01015333		
Agarophyton vermiculophyllum		GILD01018007		
Asparagopsis taxiformis	JAAEFF010000038			
Betaphycus philippinensis		GFKP01010377		
Chondrus crispus	CAKH01002526			
Cyanidioschyzon merolae	AP006496			
Dumontia simplex		GFKQ01011312		
Eucheuma denticulatum		GGYH01000258		
Galdieria sulphuraria	ADNM02000015			
Gracilaria chilensis		GEZJ01000507		
Gracilariopsis lemaneiformis	NFUL01011477			
Kappaphycus alvarezii	NADL03000517			
Laurencia pacifica		GFZU01090424		
Neopyropia yezoensis	WMLA01000001			
Porphyra umbilicalis	MXAK01000145			
Porphyridium purpureum	AROW01000939			
Bicosoecida sp		HBFS01010375		
Cafeteria roenbergensis	VLTO01000003	HBET01003355		
Halocafeteria seosinensis	LVLI01000290	GECG01001625		
Capsaspora owczarzaki	NW_011887305		NW_011887292	
Filasterea sp		GIQG01032363		GIQG01057006

Amoebidium parasiticum		GAKF01025188		GAKF01019907
Creolimax fragrantissima	MWQC01000007		MWQC01000005	
Ichthyophonus hoferi	NAOQ01000300		NAOQ01000126	
Ichthyosporea sp	PDIM01000103		PDIM01000021	
Sphaeroforma arctica	NW_014039900		BJTW01000031	
Fonticula alba			AROH01001500	
Amorphochlora amoebiformis				HBEM01008576
Bigelowiella natens	ADNK01000778	HBQC01115040	ADNK01002595	HBQJ01009950
Chlorarachnion reptans				HBKK01020031
Lotharella globosa		HBIV01002198		HBKI01038678
Partenskyella glossopodia		HBMD01025895		HBMD01029209
Plasmodiophora brassicae	CDSF01000079		SGDY01000128	
Polymyxa betae	RBZT01000146		RBZT01000418	
Spongospora subterranea	OUQQ01000806		OUQQ01000068	
Paulinella chromatophora	CP000815			
Globobulimina GloT15				GGCD01008754
Reticulomyxa filosa			ASPP01010626+ASPP01027358	
Rhizaria sp			MEDY01000012	
Asterionella formosa 1, Asterionella formosa 2	NKIB01005332, NKIB01013902			
Asterionellopsis glacialis1, Asterionellopsis glacialis2		HBNH01012793, HBNH01019278		
Chaetoceros neogracilis1, Chaetoceros neogracilis2		HBNY01019028, HBNY01009427		
Conticribra weissflogii1, Conticribra weissflogii2		HBJG01009248, HBJG01026877		
Corethron pennatum1, Corethron pennatum2		HBOD01065625, HBOD01066007		
Coscinodiscus wailesii1, Coscinodiscus wailesii2		HBJZ01015081, HBJZ01001142		
Cylindrotheca closterium1, Cylindrotheca closterium2		HBKL01033654, HBKL01022808		
Dactyliosolen fragilissimus1, Dactyliosolen fragilissimus2		HBLI01005951, (HBLI01022641, HBLI01022640)		
Ditylum brightwellii1, Ditylum brightwellii2		HBGN01008126, HBNV01029911		
Fistulifera solaris1, Fistulifera solaris2	BDSP01000252, BDSP01000123			
Fragilariopsis cylindrus 1, Fragilariopsis cylindrus 2	JGI Genome portal Fracy1|scaffold_6, JGI Genome portal Fracy1|scaffold_4			
Guinardia flaccida1, Guinardia flaccida2		HBQR01039138, HBQR01037321		

Leptocylindrus danicus		HBGY01004789, HBMO01004612		
Minidiscus sp		HBQM01043530		
Minutocellus polymorphus1, Minutocellus polymorphus2		HBJY01008881, HBJY01024220		
Navicula sp1, Navicula sp2		HBQT01029886, HBQT01034723		
Nitzschia sp1, Nitzschia sp2		GAKA01011847, GAKA01019999		
Odontella aurita		HBKQ01038465		
Phaeodactylum tricornutum 1, Phaeodactylum tricornutum 2	ABQD01000139	KR017888		
Pseudo-nitzschia multistriata, Pseudo-nitzschia fraudulenta	CVUE01001457	HBPF01082760		
Skeletonema marinoi1, Skeletonema marinoi2		HBNC01020279, HBGZ01017189		
Stephanopyxis turris1, Stephanopyxis turris2		HBLN01037939, HBLN01009941		
Synedra sp1, Synedra sp2		HBQV01032622, HBQV01003645		
Synedropsis cf. recta		HBME01008831		
Thalassiosira pseudonana 1, Thalassiosira pseudonana 2	AAFD02000005, AAFD02000023			
Tryblionella compressa1, Tryblionella compressa2		HBOK01021800, HBOK01015378		
Nannochloropsis gaditana1, Nannochloropsis gaditana2	AZIL01000274, JU963886			
Aplanochytrium kerguelense 1, Aplanochytrium kerguelense 2	JGI Genome portal Aplke1 >scaffold 11, >scaffold 48	HBSS01011156, HBSS01012715		
Aurantiochytrium sp	LNGJ01005598			
Schizochytrium sp	JTFK01000957	HBIY01011933		
Hondaea fermentalgiana	BEYU01000053			
Labyrinthula sp	JAALGZ010000022			
Thraustochytrium sp1, Thraustochytrium sp2	MUFY01001120	HBSU01007702		
Achlya hypogyna	JNBR01000082			
Albugo candida1, Albugo candida2	CAIX01000003		JZXB01001689	
Aphanomyces invadans 1, Aphanomyces invadans 2	AYTH01000282		AYTH01002484	
Bremia lactucae1, Bremia lactucae2	SHOA01000118			JP959449
Globisporangium iwayamae1, Globisporangium iwayamae2	NCVO01003235		AKYA02006742	
Hyaloperonospora arabidopsidis 1, Hyaloperonospora arabidopsidis 2	ABWE02005194		ABWE02009650	
Lagenidium giganteum	NSDO01000414			

Paralagenidium karlingii1, Paralagenidium karlingii2	PTTM01000548		PTTM01002049	
Peronospora effusa			NPIT01003874	
Phytophthora kernoviae1, Phytophthora kernoviae2, Phytophthora kernoviae3	JPWV02000001		AOFK02000004, AOFK02000199	
Phytopythium vexans1, Phytopythium vexans2	AKYC02001594		QLOC01000011	
Pilasporangium apinafurcum 1, Pilasporangium apinafurcum 2	BCKE01000032		BCKE01000061	
Plasmopara halstedii1, Plasmopara halstedii2	CCYD01002047		LFUD01001925	
Pseudoperonospora cubensis	AHJF01000270			
Pythium insidiosum 1, Pythium insidiosum 2	BCFR01000691	FX527878	BCFR01000198	
Saprolegnia parasitica 1, Saprolegnia parasitica 2	ADCG02000741		NW_012156468	
Sclerospora graminicola 1, Sclerospora graminicola 2	MIQA02010931		MIQA02005085,	
Thraustotheca clavata	JNBS01000368			
Blastocystis hominis	NW_013171834			
Aureococcus anophagefferens1, Aureococcus anophagefferens2	ACJI01000071, ACJI01000835			
Agarum clathratum		GEWO01000346		
Alaria esculenta		GEWN01000220		
Chorda filum		GEWG01000316		
Cladosiphon okamuranus 1,Cladosiphon okamuranus 2, Cladosiphon okamuranus 3	BDDF01000031, BDDF01000395, BDDF01000971			
Colpomenia sinuosa	GFKL01014802			
Costaria costata	GEWB01038989			
Cymathaere triplicata		GEWI01000258		
Desmarestia viridis1, Desmarestia viridis2		GFKF01023045, GFKF01029735		
Dictyopteris undulata		GFKK01012702		
Ecklonia radiata		GEWC01001518		
Ectocarpus siliculosus 1, Ectocarpus siliculosus 2	FN648403, FN648486			
Egregia menziesii		GEWJ01000581		
Fucus ceranoides		HACY01015303		
Ishige okamurae		GFKC01015406		
Laminaria digitata	GEWP01000642			
Lessonia nigrescens1, Lessonia nigrescens 2		GEWF01002518, GEWF01001035		
Macrocystis pyrifera	JAALFD010118001, JAALFD010011835			

Nereocystis luetkeana 1, Nereocystis luetkeana 2	GEWH01000711, GEWH01001046			
Petalonia fascia	GFKO01012852			
Pleurophycus gardneri		GEWL01000352		
Pseudochorda nagaii1, Pseudochorda nagaii2		IABM01037975, IABM01045769		
Pterygophora californica		GEWK01000252		
Punctaria latifolia		GFKD01032691		
Saccharina japonica 1, Saccharina japonica 2	JXRI01000032, JXRI01001610			
Sargassum integerrimum	GFLE01007458			
Scytosiphon dotyi	GFKN01010576			
Undaria pinnatifida1, Undaria pinnatifida2		IABO01027752, IABO01033594		
Heterococcus sp	AXNI01006842			
Tribonema minus	JAFCMP010000067			
Vaucheria litorea		HBPK01032616		
Asterochloris sp	JGI Astpho2|scaffold_0012			
Auxenochlorella protothecoides	APJO01000804	GDKF01007569		
Bathycoccus prasinos	NC_023995			
Botryococcus braunii	MVGU01000327			
Chlamydomonas reinhardtii	ABCN01004009			
Chlorella pyrenoidosa	ANZC01003881			
Coccomyxa subellipsoidea	AGSI01000012			
Dunaliella primolecta	OU611765			
Helicosporidium sp	AYPS01000749			
Klebsormidium flaccidum	BANV01000180			
Micromonas pusilla	NW_003315883			
Ostreococcus tauri	CR954202			
Picochlorum sp	JPID01000019			
Tetraselmis (Platymonas) sp	GBEZ01003863			
Ulva lactuca	GFTX01065962			
Volvox carteri	ACJH01007390			

Online Resource 3
Listing of accession numbers of entries used to derive the arginyl-tRNA synthetase sequences. Accession numbers joined by "+" represent non-overlapping contigs. The species are listed in the order given in Online Resource 1. ArgRS; arginyl-tRNA synthetase, Cyto; cytosolic, Mito; mitochondrial, TSA; transcriptome
[1]Sequence very similar to metazoan mitochondrial arginyl-tRNA synthetase. Host contamination?

Evolutionary adjustment of tRNA identity rules in Bacillariophyta for recognition by an aminoacyl-tRNA synthetase adds a facet to the origin of Diatoms
J.Mol.Evol.
Gabor L. Igloi, University of Freiburg; igloi@biologie.uni-freiburg.de
Online Resource 3
